# Typical visual search performance and atypical gaze behaviors in response to faces in Williams syndrome

**DOI:** 10.1186/s11689-016-9172-7

**Published:** 2016-10-24

**Authors:** Masahiro Hirai, Yukako Muramatsu, Seiji Mizuno, Naoko Kurahashi, Hirokazu Kurahashi, Miho Nakamura

**Affiliations:** 1 Institute for Developmental Research, Aichi Human Service Center, 713-8 Kagiya-cho, Kasugai, Aichi 480-0392 Japan; 2 Department of Pediatrics, Central Hospital, Aichi Human Service Center, 713-8 Kagiya-cho, Kasugai, Aichi 480-0392 Japan; 3 Present address: Center for Development of Advanced Medical Technology, Jichi Medical University, 3311-1 Yakushiji, Shimotsuke, Tochigi 392-0498 Japan

**Keywords:** Williams syndrome, Face perception, Attention, Eye tracking

## Abstract

**Background:**

Evidence indicates that individuals with Williams syndrome (WS) exhibit atypical attentional characteristics when viewing faces. However, the dynamics of visual attention captured by faces remain unclear, especially when explicit attentional forces are present. To clarify this, we introduced a visual search paradigm and assessed how the relative strength of visual attention captured by a face and explicit attentional control changes as search progresses.

**Methods:**

Participants (WS and controls) searched for a target (butterfly) within an array of distractors, which sometimes contained an upright face. We analyzed reaction time and location of the first fixation—which reflect the attentional profile at the initial stage—and fixation durations. These features represent aspects of attention at later stages of visual search. The strength of visual attention captured by faces and explicit attentional control (toward the butterfly) was characterized by the frequency of first fixations on a face or butterfly and on the duration of face or butterfly fixations.

**Results:**

Although reaction time was longer in all groups when faces were present, and visual attention was not dominated by faces in any group during the initial stages of the search, when faces were present, attention to faces dominated in the WS group during the later search stages. Furthermore, for the WS group, reaction time correlated with eye-movement measures at different stages of searching such that longer reaction times were associated with longer face-fixations, specifically at the initial stage of searching. Moreover, longer reaction times were associated with longer face-fixations at the later stages of searching, while shorter reaction times were associated with longer butterfly fixations.

**Conclusions:**

The relative strength of attention captured by faces in people with WS is not observed at the initial stage of searching but becomes dominant as the search progresses. Furthermore, although behavioral responses are associated with some aspects of eye movements, they are not as sensitive as eye-movement measurements themselves at detecting atypical attentional characteristics in people with WS.

## Background

Williams syndrome (WS) is a rare genetic disorder caused by the deletion of approximately 28 genes on chromosome 7, and has a prevalence ranging from 1 in 7500 to 1 in 20,000 [1, 2]. Along with its associated physical characteristics, such as dysmorphic facial features and heart defects, a unique hypersociable profile has been described [3, 4] which includes extreme interest in both familiar and unfamiliar people, both in terms of approach to strangers [3, 5–9] and prolonged staring at faces [10]. Even very young children with WS have been reported to show an atypically high interest in faces [11].

Combining eye-tracking techniques and behavioral responses has become a useful method to explore attentional profiles for individuals with WS that relate to their heightened interest in faces. For instance, Riby and Hancock [10] used a free-viewing paradigm with eye tracking to demonstrate that people with WS pay more attention to faces in social scenes than do control groups (non-verbal ability-matched and chronologically age-matched) or those with autism spectrum disorder. Similarly, another free-viewing eye-tracking study showed that people with WS fixated faces in a scene longer than did typical controls matched for non-verbal ability [12]. Additionally, a series of behavioral experiments using reaction time measures revealed that attention capture by faces, interference from faces, and face biases are typical in WS, while disengaging attention from faces happens with an atypically low frequency [13]. Thus, people with WS appear to spend an unusually long time looking at faces, most likely because of problems disengaging attention.

While eye-tracking studies can tell us how the presence of a face affects gaze behavior and when eye movements and fixations are made, most have adopted a passive, free-viewing paradigm. In contrast to free-viewing eye-tracking studies, behavioral experiments that measure reaction time give us information regarding the relative strength of visual attention captured by faces and explicit attentional control. In Langton et al. [14], participants were asked to search for a butterfly among distractors that included a face. Even though the face was unrelated to the target, reaction time was longer in face-present trials than in face-absent trials. These findings suggest that even though faces were unrelated to the task, they captured attention and slowed performance. However, when reaction time and accuracy are the only indices of performance, determining how the presence of faces captured attention and influenced performance is not possible—for example, it is unclear whether faces captured attention immediately or later during trials.

The aim of the current study was to gage the relative strengths of attention captured by a target-unrelated face and explicit attentional control toward a target butterfly during the initial and later stages of directed searches made by individuals with WS. We adopted the experimental paradigm developed by Langton et al. [14] that included manual responses and eye tracking so that analyses could be made during the different stages of the visual search. Participants were asked to search for a target butterfly among six objects that were placed in a circular arrangement. This arrangement ensured objects were equidistant from the center of the screen. In some trials, a distractor was replaced with a face, which allowed the relative strength of goal-directed searching to be compared with stimulus (i.e., face- or non-face)-driven attention. Riby et al. [13] used the same paradigm but with a different arrangement of stimuli—each stimulus was displayed in a grid. They only measured reaction time and found that people with WS showed the same level of face-induced interference as typical controls. However, because of their paradigm design, stimuli differed in distance from the central fixation trial-by-trial, which could have affected reaction time. To best quantify the degree to which faces interfere with the task, stimuli should be equidistant from the center so that the only factors affecting peripheral attention are the identities of the stimuli.

We analyzed the location of the first fixation and the subsequent fixation durations to reveal how the dominating force in visual attention (the target-unrelated face or the explicit attentional control toward a target) change over time. Because the initial fixation is rapid, it reflects the attentional profile at the early stages of visual search. Fixation duration shows how much time participants gazed at each item during the search. Therefore, it reflects the attentional profile at later stages of processing. By using these two eye movement measures, we can assess the relative strengths of visual attention captured by a target-unrelated face and explicit attentional control toward a target at each stage of the search. In the early stage, if visual attention captured by a target-unrelated face is relatively stronger than that toward the target (i.e., butterfly), the first fixation should land more frequently on the face than on objects or the butterfly. However, if the reverse is true, the first fixation should land on the butterfly more frequently than on objects or faces. In the later stage of searching, if visual attention captured by a target-unrelated face is relatively stronger than explicit attentional control toward the butterfly, fixation-duration proportion for face should be longer than that for object or butterfly. However, if the reverse is true, fixation-duration proportion for butterfly should be longer than that for face.

We predicted several outcomes. First, we predicted that reaction time would be longer in face-present trials than in face-absent trials for both controls and people with WS, as this has been seen in another study [13]. Second, we predicted that explicit attentional control toward a target would be relatively dominant at the early stage of searching because even in free-viewing paradigms, initial attention is not significantly directed toward faces [12]. Third, we predicted that for individuals with WS, once they fixated on a face, they would have difficulty disengaging from it at the later stages of the search because of their difficulty disengaging attention [13]. Fourth, we predicted that if gaze behaviors at different stages of visual search are tightly linked to reaction time, reaction time would be significantly affected by the eye-movement indices. More precisely, we predicted that explicit attentional control would be dominant at the initial stage of searching. As a result, reaction time would negatively correlate with the frequency of initial butterfly-fixations. However, if visual attention captured by faces is stronger than explicit attentional control at the initial stage of searching, then reaction time would be positively correlated with the frequency of the first fixation toward faces. Additionally, if visual attention captured by a face is stronger at the later stages of searching, reaction time would positively correlate with the duration of face-fixations. Otherwise, it would negatively correlate with the duration of butterfly-fixations.

## Methods

### Participants

Twenty-four individuals with WS participated in the experiment. Nineteen people with WS were recruited from our institute, and five were recruited through the Williams Syndrome Association in the Chubu region of Japan (Elfin Chubu, Nagoya, Japan). All participants had been previously diagnosed phenotypically by clinicians, with subsequent confirmation using fluorescence in situ hybridization testing. All inclusion and exclusion methods were based on other similar previous studies [10, 12, 15]. Data from three individuals with WS were excluded, the first because the percentage of correct trials was below chance level, and the two others because of technical problems with the eye tracker. Thus, data from 21 individuals with WS were analyzed (Table 1; 11 males and 10 females; age range 6;11–33;5 years; mean age 16.3 years). Mental age was measured using Raven’s Colored Progressive Matrices test (RCPM) [16, 17].

**Table 1:** Participant information

Group	N (F/M)	Chronological age, mean (years), range (years; months)	RCPM score
WS	21^a^ (10/11)	16.2 ± 7.1 (6; 11–33; 5)	18.3 ± 5.0 (13–33)
MA	21 (13/8)	5.8 ± 0.7 (4; 1–7; 0)	20.3 ± 4.4 (10–26)
CA	21^b^ (10/11)	15.8 ± 6.4 (6; 11–29; 4)	N/A

Mean ± SD

^a^Seven individuals with WS were adult (above 18 years old) (4 females and 3 males; mean age 24.5 years) and 14 individuals with WS were children (6 females and 8 males; mean age 12.0 years)

^b^Nine individuals in the CA group were adult (5 females and 4 males; mean age 22.3 years) and 12 individuals in the CA group were children (5 females and 7 males; mean age 11.0 years)

Forty-two children, adolescents, and adults with typical development from nearby elementary schools, a junior high school, a high school, and universities were recruited as control participants (Table 1). All control participants were screened for history of neurological or psychiatric disorder, developmental disorder, or learning difficulty, and no such issues were reported. All participants had normal or corrected-to- normal vision. For the mental age-matched (MA) group, 21 children (8 males and 13 females; age range 4;1–7;0 years; mean age 5.8 years) were recruited, and as in previous studies [12, 13] were matched to the WS group based on non-verbal ability as measured by the RCPM. For the chronological age-matched (CA) group, 21 individuals were recruited and individually matched for age to participants in the WS group (11 males and 10 females; age range 6;11–29;4 years, mean age 15.8 years). There were no significant group differences in RCPM score (WS mean 18.3, MA mean 20.3, *p* = 0.18) or age (WS mean 16.2 years, CA mean 15.8 years, *p* = 0.87).

All the children, their parents, and the adult participants provided informed consent to take part in the study, which was approved by the ethics committee at the Institute for Developmental Research at the Aichi Human Service Center (Reference Number: 04-08).

### Stimuli and apparatus

The experiment was conducted using a computer (HP Pavilion Desktop, h8-1060jp) using the Tobii Studio and E-prime 2.0 software (Psychology Software Tools, Inc., PA, USA) and with the E-prime extension for Tobii (Tobii, Inc., Stockholm, Sweden). Stimuli were presented on a 24-in. LCD color monitor (Iiyama, PLE2407HDS), placed approximately 60 cm from the observer.

**Fig. 1:**
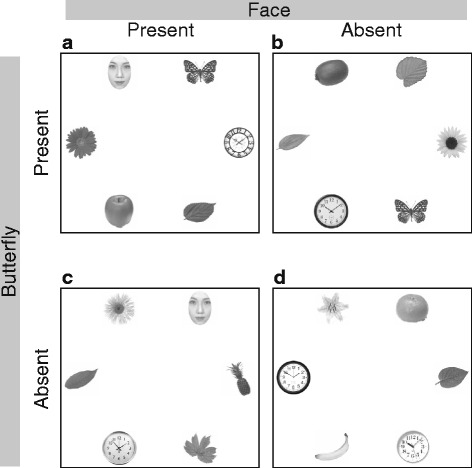
Experimental conditions. **a** Face-present/butterfly-present. **b** Face-absent/butterfly-present. **c** Face-present/butterfly-absent. **d** Face-absent/butterfly-absent. Participants were instructed to search for a butterfly and the experimenter did not mention that there would be any face stimuli

As in Langton et al. [14], six images of objects, including a face, were displayed in a circular array (Fig. 1). The objects consisted of gray-scale shapes that were sized to fit within a 3.5° × 3.5° square. The averaged luminance for all objects was equated using the SHINE toolbox [18]. The center of each object was located at approximately 15° of visual angle from the central fixation point of the display.

Because we wanted to determine how a target-unrelated face stimulus could modulate gaze behavior when both a target and a face were present, we included four conditions based on manipulating two factors: target (present vs. absent) and face (present vs. absent). For each experimental condition, 30 arrays were created, which yielded 120 trials. The target objects were six different pictures of butterflies. The face distractors were obtained from a commercially available database (ATR, Kyoto, Japan), and comprised eight different upright faces (four male and four female), all of which had neutral expressions and were cropped to remove external features, such as hair and ears. The other distractors were varied exemplars from the categories of fruit, flowers, leaves, clocks, and houseplants. Non-face distractor locations in each array were filled with different objects selected at random from these five object categories. In the butterfly-present /face-present condition (Fig. 1a), a target butterfly appeared at one of the six possible locations, and a face appeared at one of the remaining five. Each trial had a unique butterfly–face pairing. Target-present/face-absent arrays (Fig. 1b) were created by replacing the face in each array with an object that was randomly selected from one of the five object categories, with the constraint that an array could not contain two identical pictures.

For the target-absent/face-present condition (Fig. 1c), a face was located at one of the six possible locations and the remaining distractor locations in each array were filled with objects selected at random from the five object categories. For the target-absent/face-absent condition (Fig. 1d), the array was created by selecting six different objects from the five categories.

Behavioral responses were reported via a custom-made response box with two large buttons. Eye movements were recorded using the Tobii X60 eye-tracking system. The eye-tracking system was completely non-invasive, and it was not necessary to artificially constrain head or body movements. The system tracked both eyes with an accuracy of 0.5° and a sampling rate of 60 Hz. The eye tracker was calibrated for each participant using a five-point calibration of each eye.

### Task and procedure

**Fig. 2:**
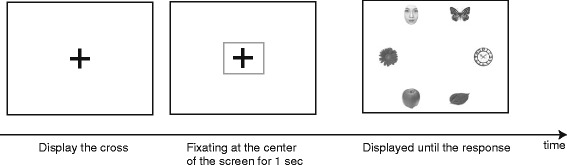
Experimental procedure. A fixation cross was displayed at the center of the screen. If the participant fixated on it for 1 s, the stimulus array would be displayed. After pressing a response button, the stimulus array disappeared

To record reliable eye-movement data during each trial in younger children and in people with WS, the stimulus array was not presented until participants fixated a cross at the center of the screen for 1 s (Fig. 2). Participants were asked to judge as quickly and accurately as possible whether a butterfly was present in each array, and to make their responses by pressing one of the two buttons on the response box. Half of the participants were asked to using their left hand and press the left button if they saw the butterfly and to press the right button if they did not. The other half of the participants were asked to use their right hand and were given the opposite instructions. No feedback was given to participants. Additionally, participants were not told that faces would be appearing on the screen, and the experimenter emphasized that they should concentrate on finding the butterfly. Before completing the actual experiment, participants performed 12 practice trials.

The experiment consisted of three sessions, each with 40 trials (four conditions × ten trials per condition). Trials were randomized within a session and the order of sessions was randomized. Between sessions, participants were given a 1- to 2-min break, if required. The entire duration of the instruction, practice, calibration, and actual experiment was about 15–20 min.

### Data analysis

We analyzed behavioral responses and eye movements. For behavioral responses, both the reaction time and performance accuracy (percent correct) were analyzed using mixed-design repeated measures analysis of variance (ANOVAs). Tukey’s HSD was applied for multiple comparisons across groups. A three-way ANOVA was applied to the reaction time and performance accuracy. Group was used as a between-participant factor (WS, MA, and CA groups), and target (present and absent) and face (present and absent) were used as within-participant factors. The index of face distraction [19] was defined as the difference between reaction time in the face-present condition and that in the face-absent condition when the target was present. A one-way ANOVA was applied to the index, and group was used as a between-participant factor (WS, MA, and CA groups).

For gaze behaviors, we first assessed basic oculomotor measures such as percent of time spent making saccades, saccade duration, percent of time spent fixating onscreen, average fixation duration, and amount of lost data. Saccades were defined by the *I*-*V* filter originally equipped with Tobii Studio. A one-way ANOVA was applied to all gaze behaviors, and group was used as a between-participant factor (WS, MA, and CA groups).

We also assessed task-related eye-movement indices that might relate to how a target-unrelated face could modulate gaze behavior. Six areas of interest were defined by polygons that encompassed the whole image at each of the six stimulus locations. This allowed us to determine the first fixation [20] and the fixation durations [10], which could then be used to evaluate the relative strengths of visual attention captured by target-unrelated faces and explicit attentional control toward the butterfly at different stages of searching. The first-fixation proportion (see below) was the frequency with which participants fixated on an item (face, butterfly, or objects) at the end of the first saccade. Therefore, it can characterize the relative strengths of visual attention captured by faces and explicit attentional control (toward butterfly) attention at the initial stage of searching. The fixation durations indicate how long a participant gazed at each item during the experiment. Therefore, they can characterize the relative dominance of visual attention captured by faces and explicit attentional control toward a target at the later stages of searching.

Because the time spent searching for a target varied across participants and groups, it was necessary to calculate first-fixation and fixation-duration proportions based on the total numbers of first fixations and fixation durations, respectively (termed “first-fixation proportion” and “fixation-duration proportion”). The first-fixation proportion was defined as the number of first fixations made to an item (butterfly, face, or object) divided by the total number of the trials. This measure can be used to assess which items received attentional priority in a scene. The fixation-duration proportion was defined as the amount of time spent looking at each type of stimulus during a trial divided by the total duration of fixations for that trial. Both proportions were further modified for the face-present and butterfly-present conditions by dividing the value by the number of non-face distractors (i.e., four items). A two-way ANOVA was applied to the data for both proportions. Group was used as a between-participant factor (WS, MA, and CA groups), and the items (face, butterfly, and objects) were used as within-participant factors. Tukey’s HSD was applied for multiple comparisons. We considered statistical significance to be *p* < 0.05.

## Results

### Reaction times (RTs)

**Fig. 3:**
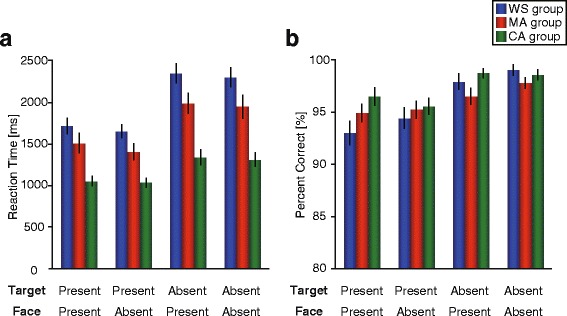
Behavioral performance. Reaction times and performance accuracy for the four conditions (Target present/face present, target present/face absent, target absent/face present, target absent/face absent) are shown. **a** Mean reaction times. (**b**) Mean performance accuracy. Error bars indicate standard error of the mean (S.E.M). WS: Williams syndrome group, MA: mental age-matched group, CA: chronological age-matched group

For reaction time (Fig. 3a), we found significant main effects of group (*F*_2, 60_ = 19.8, *p* < 0.01, *η*_*p*_^*2*^ = 0.40), target (*F*_1, 60_ = 250.7, *p* < 0.01, *η*_*p*_^*2*^ = 0.81), and face (*F*_1, 60_ = 11.5, *p* < 0.01, *η*_*p*_^*2*^ = 0.16). In terms of the effect of group, reaction time was faster for the CA group than for the MA group or the WS group (WS 2000 ms, MA 1708 ms, and CA 1182 ms). For target, reaction time was faster for the target-present condition than for target-absent condition (target present 1392 ms, target absent 1868 ms). For face, reaction time was slower in the face-present condition than in the face-absent condition (face present 1656 ms, face absent 1604 ms).

Furthermore, the group × target interaction was significant (*F*_2, 60_ = 12.0, *p* < 0.01, *η*_*p*_^*2*^ = 0.29). To explore the nature of the interaction, tests on the simple main effects were performed. The simple main effect of group for both target present (*F*_2, 120_ = 11.2, *p* < 0.01, *η*_*p*_^*2*^ = 0.16) and target absent (*F*_2, 120_ = 27.2, *p* < 0.01, *η*_*p*_^*2*^ = 0.31) were significant. For the target-present condition, a Tukey post hoc test showed that reaction time was significantly longer for the target-present condition in the WS and MA groups than in the CA group (vs. WS *p* < 0.01; 1681.7 vs. 1041.6 ms; vs. MA *p* < 0.01; 1453.5 vs. 1041.6 ms) and that the WS and MA groups did not differ significantly (*p* = 0.09). Conversely, we found significant differences across all groups for the target-absent condition (all *ps* < 0.05; WS 2318.4, MA 1963.3, CA 1322.4 ms). All main effects of target within the WS (*F*_1, 60_ = 149.7, *p* < 0.01, *η*_*p*_^*2*^ = 0.72), MA (*F*_1, 60_ = 95.9, *p* < 0.01, *η*_*p*_^*2*^ = 0.62), and CA groups (*F*_1 60_ = 29.1, *p* < 0.01, *η*_*p*_^*2*^ = 0.33) were significant. This indicates that reaction time was significantly faster in the target-present condition than in the target-absent condition for all groups (WS 1681.7 vs. 2318.4 ms, MA 1453.5 vs. 1963.3 ms, CA 1041.6 vs. 1322.4 ms). Other interactions, such as the group × face interaction (*F*_2, 60_ = 0.96, *p* = 0.39) and the group × target × face interaction (*F*_2, 60_ = 0.61, *p* = 0.55, *η*_*p*_^*2*^ = 0.02) did not reach statistical significance. Therefore, the groups differed in their reaction times, as the CA group had faster reaction times than the WS or MA groups. However, they showed similar patterns, as reaction times for all groups were faster in target-present trials than in target-absent trials, and were slower in face-present trials than in face-absent trials. Although the WS group and the MA performed similarly in the target-present conditions (and both worse than the CA group), the WS group performed worse than the MA group in the target-absent condition.

As for the index of face distraction, we found no significant effect of group (*F*_2, 60_ = 1.90, *p* = 0.16, *η*_*p*_^*2*^ = 0.06), indicating that appearance of the face in the target-present trials affected each group similarly.

### Accuracy

For performance accuracy (Fig. 3b), we found a significant main effect of target (*F*_1, 60_ = 65.0, *p* < 0.01, *η*_*p*_^*2*^ = 0.52), but no main effect of group (*F*_2, 60_ = 1.27, *p* = 0.11, *η*_*p*_^*2*^ = 0.07) or face (*F*_1, 60_ = 1.48, *p* = 0.23, *η*_*p*_^*2*^ = 0.02). The main effect of target showed that overall, performance accuracy was higher when the target was absent than when it was present (96.5 vs. 93.5 %). Furthermore, there was a significant group × target interaction (*F*_1, 60_ = 4.44, *p* = 0.02, *η*_*p*_^*2*^ = 0.13). To explore the nature of this interaction, tests on the main effects were performed. The main effect of target within each group was significant (WS group: *F*_1, 60_ = 49.6, *p* < 0.01, *η*_*p*_^*2*^ = 0.45; MA group: *F*_1, 60_ = 9.3, *p* < 0.01, *η*_*p*_^*2*^ = 0.13; CA group: *F*_1, 60_ = 15.0, *p* < 0.01, *η*_*p*_^*2*^ = 0.20). This indicates that performance accuracy was significantly higher in the butterfly-absent trials than in the butterfly-present trials for all groups (all *ps* < 0.01). Furthermore, the main effect of group was significant within target-present trials (*F*_2, 120_ = 4.0, *p* < 0.05, *η*_*p*_^*2*^ = 0.06), but not within target-absent trials (*F*_2, 120_ = 2.1, *p* = 0.13, *η*_*p*_^*2*^ = 0.03). This indicates that accuracy was significantly higher in the CA group than in the WS group for the target-present condition (*p* = 0.02). Other comparisons did not show any significance (all *ps* > 0.10). For the group differences in target-absent trials, no significant effect was observed (*p* = 0.13). Therefore, all groups were equally more accurate in trials in which the butterfly was absent than when it was present. In trials in which the butterfly was present, the CA group was more accurate than the WS group, and there were no differences between the MA group and the CA or WS groups.

### Gaze behavior (basic oculomotor measures)

We first assessed eight basic oculomotor measures, four based in saccades and four based on fixations: (1) Percent of time spent making saccades: The main effect of group was significant (*F*_2, 60_ = 18.5, *p* < 0.01, *η*_*p*_^*2*^ = 0.38), and a Tukey post hoc test showed that the WS group spent significantly more time making eye movements (35.8 %) than either the MA (23.9 %) or CA (25.9 %) groups (*ps* < 0.01). No significant difference was observed between the MA and CA groups (*p* = 0.34). (2) Total saccade duration: The main effect of group was significant (*F*_2, 60_ = 27.9, *p* < 0.01, *η*_*p*_^*2*^ = 0.48), and a Tukey post hoc test showed that the time spent making saccades in the WS group (mean 81.3 s) was significantly longer than that in either the MA (mean 49.3 s) or CA (mean 37.4 s) groups (*ps* < 0.01). No significant difference was observed between the MA and CA groups (*p* = 0.06). (3) Number of saccades: The main effect of group was significant (*F*_2, 60_ = 11.5, *p* < 0.01, *η*_*p*_^*2*^ = 0.28), and a Tukey post hoc test showed that the WS group made significantly more saccades (mean 1847.4) than either the MA (*p* < 0.05; mean 1536.3) or CA (*p* < 0.01; mean 1210.9) groups. Moreover, the MA group made significantly more saccades than the CA group (*p* < 0.05). (4) Mean saccade duration: The main effect of group was significant (*F*_2, 60_ = 11.2, *p* < 0.01, *η*_*p*_^*2*^ = 0.27), and a Tukey post hoc test showed that saccade duration were significantly longer on average in the WS group (mean 0.045 s) than in either the MA (mean 0.032 s) or CA (mean 0.031 s) groups (*ps* < 0.01). No significant difference was observed between the MA and CA groups (*p* = 0.85). (5) Percent of time spent fixating: The main effect of group was significant (*F*_2, 60_ = 18.5, *p* < 0.01, *η*_*p*_^*2*^ = 0.38), and a Tukey post hoc test showed that it was significantly shorter in the WS group (64.1 %) than in either the MA (76.1 %) or CA (74.1 %) groups (*ps* < 0.01). No significant difference was observed between the WS and CA groups (*p* = 0.34). (6) Total fixation duration: The main effect of group was significant (*F*_2, 60_ = 8.9, *p* < 0.01, *η*_*p*_^*2*^ = 0.23), and a Tukey post hoc test showed the WS (144.4 s) and MA (158.5 s) groups spent significantly less time fixating than the CA group (108.2 s; *ps* < 0.01). No significant difference was observed between the MA and the WS group (*p* = 0.26). (7) Number of fixations: The main effect of Group was significant (*F*_2, 60_ = 8.9, *p* < 0.01, *η*_*p*_^*2*^ = 0.23), and a Tukey post hoc test showed that the CA group made fewer eye movements (591.6) than either the MA (776.3) or WS (788.7) groups (*ps* < 0.01). No significant difference was observed between the WS and MA groups (*p* = 0.81). (8) Mean fixation duration: We did not find a significant main effect of group (*F*_2, 60_ = 2.2, *p* = 0.12, *η*_*p*_^*2*^ = 0.07; CA: 0.184 s, MA 0.201 s, WS: 0.186 s).

We analyzed the amount of lost data during the trial period across the entire experiment, and found a significant main effect of group (*F*_2, 60_ = 9.8, *p* < 0.01, *η*_*p*_^*2*^ = 0.25), and a Tukey post hoc test showed that it was significantly smaller in both the MA and CA groups than in the WS group (*ps* < 0.01; CA 4.7 %, MA 3.7 %, WS 12.6 %). We further confirmed whether our current experimental paradigm allowed us to record reliable data from all participants during experimental trials (excluding the rest period). We determined the number of trials available in each group for analyzing the first-fixation proportion, and found no significant differences across groups (CA 28.3 ± 2.7 trials, MA 29.5 ± 1.3 trials, WS 29.3 ± 2.3 trials; *F*_2,60_ = 1.68, *p* = 0.19, *η*_*p*_^*2*^ = 0.001).

### Gaze behavior

As the aim of the current study was to gage the relative strengths of attention captured by a target-unrelated face and explicit attentional control toward a target butterfly during the initial and later stages of directed searches, we focused on only the face-present and target-present conditions. We compared the first-fixation proportion and fixation-duration proportion between the groups and across task conditions.

### First-fixation proportion (attentional profile at initial stage of searching)

We presumed that if visual attention captured by target-unrelated faces was stronger than explicit attentional control toward a target at the initial stage of the search, then the frequency of the first-fixation proportion for face should be higher. However, if explicit attentional control toward a target was stronger, the proportion for butterfly would be higher.

**Fig. 4:**
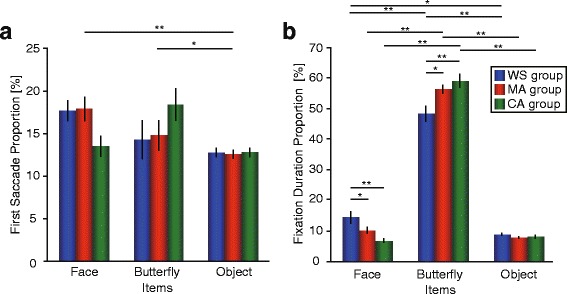
Gaze behavior. **a** First-fixation proportion and (**b**) fixation-duration proportion. Error bars indicate standard error of the mean (SEM)

We found a significant main effect of item (*F*_2, 120_ = 4.83, *p* < 0.01, *η*_*p*_^*2*^ = 0.08) on the first-fixation proportion (Fig. 4a). Subsequent Tukey post hoc analyses revealed that the first fixation was more frequently focused on butterflies and faces than on other objects (butterfly vs. object 19.1 vs. 15.3 % (*p* < 0.05), face vs. object 19.7 vs. 15.3 % (*p* < 0.01)). The group × item interaction approached significance (*F*_4, 120_ = 2.31, *p* = 0.07, *η*_*p*_^*2*^ = 0.07).

### Fixation-duration proportion (attentional profile at later stages of searching)

In a similar vein, we presumed that if visual attention captured by target-unrelated faces was stronger than explicit attentional control toward a target at later stages of the search, then the duration of face-fixations would be higher than that of other fixations. However, if explicit attentional control toward a target was stronger, butterfly-fixations would be the longest.

We found a significant main effect of items (*F*_2, 120_ = 773.08, *p* < 0.01, *η*_*p*_^*2*^ = 0.93) on the fixation-duration proportion (Fig. 4b), but the main effect of group was not significant (*F*_2, 120_ = 1.72, *p* = 0.19, *η*_*p*_^*2*^ = 0.08). The subsequent Tukey post hoc analysis revealed that the butterflies were fixated significantly longer than faces (*p* < 0.01) or objects (*p* < 0.01). Furthermore, the group × items interaction was significant (*F*_4, 120_ = 8.64, *p* < 0.01, *η*_*p*_^*2*^ = 0.22). To explore the nature of the interaction, tests on the main effects were performed. While the main effect of group was significant within both face (*F*_2, 180_ = 7.62, *p* < 0.01, *η*_*p*_^*2*^ = 0.08) and butterfly (*F*_2, 180_ = 16.5, *p* < 0.01, *η*_*p*_^*2*^ = 0.16), it did not reach statistical significance for object (*F*_2, 180_ = 0.13, *p* = 0.87, *η*_*p*_^*2*^ = 0.001). For face, a Tukey post hoc test showed that while fixation duration was significantly longer for the WS group than for the CA (*p* < 0.01) or MA groups (*p* < 0.05), it did not differ between the MA and CA groups (*p* = 0.09). For butterfly, while fixation duration was significantly shorter in the WS group than in the CA (*p* < 0.01) or MA (*p* < 0.01) groups, it did not differ between the MA and CA groups (*p* = 0.19). The main effect of Item was significant within all groups (WS: *F*_2, 120_ = 171.75, *p* < 0.01, *η*_*p*_^*2*^ = 0.74; MA: *F*_2, 120_ = 282.8, *p* < 0.01, *η*_*p*_^*2*^ = 0.82; CA: *F*_2, 120_ = 335.8, *p* < 0.01, *η*_*p*_^*2*^ = 0.85). For the WS group, a Tukey post hoc test showed that the duration of face-fixations was significantly longer than that of object-fixations (*p* = 0.02), and that the duration of butterfly-fixations was significantly longer than that of face-fixations (*p* < 0.01) or object-fixations (*p* < 0.01). For both MA and CA groups, a Tukey post hoc test showed that the duration of face-fixations was not significantly longer than that of object-fixations (MA group: *p* = 0.33, CA group: *p* = 0.60), while the butterfly-fixations were significantly longer than face-fixations (MA group: *p* < 0.01, CA group: *p* < 0.01) or object-fixations (MA group: *p* < 0.01, CA group: *p* < 0.01).

### The relationship between the behavioral responses and gaze behavior

**Fig. 5:**
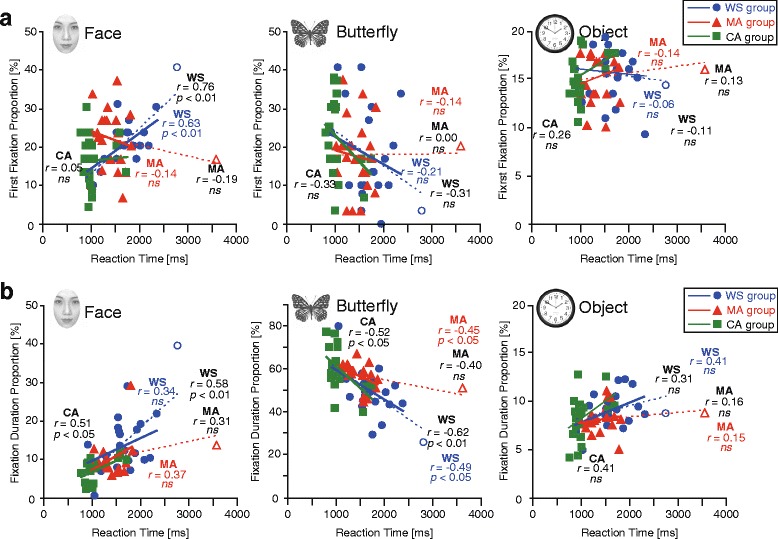
Relationship between reaction time and gaze behavior. Relationship between reaction time and the first-fixation (**a**) and the fixation-duration (**b**) ratios for the face, butterfly, and object (WS, MA, and CA groups). *WS* Williams syndrome group, *MA* mental age-matched group, *CA* chronological age-matched group. *Open circles* indicate outliers. The *dashed line* indicates the regression analysis including outliers and the *solid line* is the regression line excluding outliers. *Colored* and *black* values indicate the correlation excluding and including outliers, respectively. Note the different y-axis ranges for parts A and B

We sought between broad measures (reaction time and performance accuracies) that probe the entire visual search process, and finer measures (eye movements) that can probe the process at different stages. To assess the relationship between the broad and fine measures at the initial stage of searching, we assessed the correlation between reaction time and the first-fixation proportion (Fig. 5a). We found a significant positive correlation between these measures for faces only in the WS group (WS: *r* = 0.76, *p* < 0.01; MA: *r* = −0.19, CA: *r* = 0.05). We did not find any significant correlations in these measures for butterflies or the other objects (Fig. 5b). Inspection of the scatterplots indicated that some correlations may have been affected by outliers. Therefore, we removed the outliers (>2 SD of mean reaction time in each group; one WS and one MA participant) and recalculated the correlations. As before, we found a significant correlation between reaction time and first-fixation proportion for face only in the WS group (WS: *r* = 0.63, *p* < 0.01; MA: *r* = −0.14, CA: *r* = 0.05). We did not find any significant correlations in these measures for butterfly or the other objects (Fig. 5b).

To assess the relationship between the broad and fine measures at the later stages of searching, we calculated the correlation between reaction time and the fixation-duration proportion for each item (Fig. 5b). We found significant positive correlations between these measures for faces (WS: *r* = 0.58, *p* < 0.01, CA: *r* = 0.51, *p* < 0.05), and opposite correlations for butterflies in both the WS and CA groups (WS: *r* = −0.62, *p* < 0.01, CA: *r* = −0.52, *p* < 0.05). When we removed the data from the two participants, the correlation between reaction time and fixation-duration proportion for face was diminished (*r* = 0.34) in the WS group, but the relationship was preserved in both MA and CA groups (MA: *r* = 0.37, CA: *r* = 0.51, *p* < 0.05). The correlation between reaction time and fixation-duration proportion for butterfly was significant in all groups (WS: *r* = −0.48, *p* < 0.05, MA: *r* = −0.45, *p* < 0.05, CA: *r* = −0.52, *p* < 0.05). We did not find any significant correlation in any group between reaction time and fixation-duration proportion for objects.

The relationships between accuracy and the two proportions was also assessed (Fig. 5a). We did not find any significant correlations with the first-fixation proportion (WS: *r* = −0.29, MA: *r* = 0.20, CA: *r* = 0.00) or with the fixation-duration proportion (WS: *r* = −0.35, MA: *r* = 0.15, CA: *r* = 0.35). As in the original analysis, when we removed the two participants with outlier data, we did not find any significant correlation.

### The relationship between the RCPM scores and gaze behavior

**Fig. 6:**
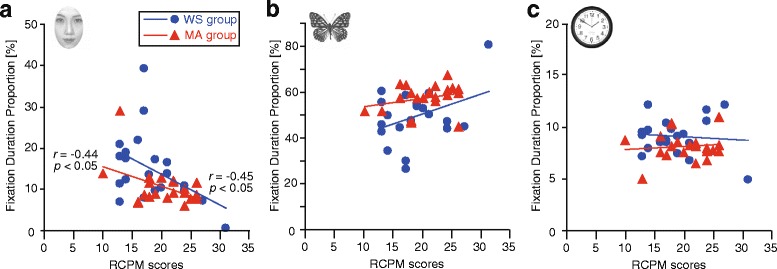
Relationship between the RCPM score and the fixation-duration proportion. **a** Face, **b** butterfly, and **c** object stimuli for the target-present and face-present conditions (WS and MA groups). We found a significant negative correlation between RCPM score and the fixation-duration proportion for the target-unrelated face stimuli, but not for the butterfly or object stimuli. *WS* Williams syndrome group, *MA* mental age-matched group, *CA* chronological age-matched group

We also assessed the correlation between age, RCPM scores, first-fixation proportion, and fixation-duration proportion for each item. For both the WS and MA groups, we observed significant negative correlations between RCPM scores and the fixation-duration proportion for face stimuli (WS: *r* = −0.45, *p* < 0.05, MA: *r* = −0.44, *p* < 0.05, Fig. 6) but not between chronological age and the fixation-duration proportion. No other significant correlations were observed.

## Discussion

This study was designed to reveal how the dynamics of explicit attentional control and visual attention captured by faces change during a visual search for people with WS when faces are presented as distracting objects. To do so, we introduced the experimental paradigm developed by Langton and colleagues [14] and simultaneously measured manual responses and eye movements. We found that a broad measure like reaction time, which reflects the duration of the entire searching process, was longer in all groups when faces were present than when they were not. However, we found that finer measures derived from eye positions could be used to discern dynamic aspects of allocating attention during the search and differences between controls and individuals with WS. The first fixation, which probes the attentional profile at the initial stage of searching, showed no group differences. However, fixation duration revealed that visual attention captured by faces was stronger than explicit attentional control toward a target in individuals with WS, but not controls, during the later search stages. Additionally, we found a significant correlation between reaction time and the two fixation measures at early and later stages of searching in both CA and WS groups.

A previous series of behavioral experiments suggested that individuals with WS are typical in terms of attention capture and engagement in faces, but take longer to disengage from them [13]. The study found that reaction times for the same groups that we employed (WS, MA, and CA) were equally affected by the presence of face stimuli (note that the arrangement of stimuli was different from that in the current study). Consistent with their findings, we also observed the interference effect of faces in all groups. In our study, despite group differences in reaction times (the CA group reacted faster than the WS or MA groups), faces had the same patterns of effects in all groups; reaction times were slower when faces were present than when they were absent. However, the degree to which targets affected reaction times did vary by group. While reaction times in both the WS and MA groups were similar in the target-present condition, they were longer in the WS group when targets were absent. Furthermore, when a face and a target were displayed at the same time, both reaction time and accuracy in individuals with WS were comparable with typical controls, suggesting that neither measure was sensitive enough to capture group-differences in response to the distracting faces. Regarding accuracy, all groups were more accurate when the butterfly was absent than when it was present. In trials in which the butterfly was present, the CA group was more accurate than the WS group, although there were no differences between the MA group and the CA or WS groups.

For the basic oculomotor measures during the experiment, the percentage of time spent making saccades, total saccade duration, saccade count, and mean saccade duration were significantly longer/larger in the WS group than in either the CA or MA groups. Furthermore, the opposite relationships were observed in the percent of time spent fixating and the total fixation duration. This shows that basic oculomotor measures in the WS group were somewhat different from controls, which could be partly because the overall reaction times in the WS group were significantly longer than those in the control groups.

For the more detailed analysis, we used the first-fixation and fixation-duration proportions. The first fixation reflects the attentional profile at the initial stage of searching and did not differ across groups. In contrast, fixation durations reflect the attentional profile at the later stages of searching. In particular, it can reflect attentional disengagement resulting from a combination of visual attention captured by the target-unrelated face and explicit attentional control toward the butterfly. As a result, the fixation-duration proportion was significantly longer in individuals with WS when saccades landed on faces. This also contrasts with the results for the manual responses. In other free-viewing eye-tracking studies, individuals with WS fixated longer on the eyes of human faces compared with typical controls [10]. Moreover, while the time required to detect a hidden face did not differ between participants with WS and typical controls, those with WS fixated longer on the embedded faces [12]. This exaggerated fixation on faces has also been reported in several case reports (e.g., [21]). Furthermore, an atypical visual search strategy in individuals with WS has even been reported when using geometric shapes [22]. As we did not find group differences in the first fixation, the visual attention captured by faces was not likely stronger than explicit attentional control toward a target at the initial stage of the visual search. However, at later stages of searching, visual attention captured by a face dominated in individuals with WS. The current findings seem to be consistent with a previous reaction time study that shows the problem that individuals with WS have with disengaging faces [13].

Both behavioral responses and eye movements were simultaneously measured in the experiment, and the visual stimulus was displayed only after participants had fixated on the center of the screen for 1 s. Therefore, these experimental settings and procedures ensured that the initial eye position was at the center of the screen, enabling us to collect reliable visual search data from all participants, including young children, with and without WS. By introducing this method, we found that behavioral measures such as reaction time and accuracy were relatively typical in WS individuals, but that gaze behavior was atypical. This method also allowed us to find relationships between these measures, considering the entire search process and their relationship with eye movements, and considering early and late stages separately. In the WS group, when all participants were included in the analysis, both first-fixation and fixation-duration proportions for face were positively correlated with reaction time, revealing the subtle irregularity in visual attention captured by faces for these people. Furthermore, the fixation-duration proportion, but not the first-fixation proportion for butterfly, was negatively correlated with reaction time, indicating an effect of explicit attentional control. These findings indicate that when visual attention captured by a face is stronger than explicit attentional control toward a target at all stages of searching, the reaction time can be prolonged. Furthermore, when explicit attentional control dominates later stages of searching, the reaction time can be shortened. We found a similar relationship at the later stages of searching in the CA group but not in the MA group. As the MA group was rather homogeneous in terms of age compared with both CA and WS groups, reaction time was less variable than those groups. This could be why we did not find significant correlations between reaction time and eye movements in the MA group. Thus, the differences between typical controls and WS individuals are subtle, and were only found when we combined broad behavioral measures with a finer analysis that could distinguish initial and later stages of searching. However, as we have shown with additional analysis, the reaction times of two participants (one WS and one MA) were considerably longer than those of other participants (>2 SD of mean reaction time in each group), and we have therefore removed their data from the analysis. While the results for first-fixation proportion remained unchanged, those for fixation-duration proportion were slightly different. Specifically, the correlation between the reaction time and fixation-duration proportion for face was reduced (*r* = 0.34) in the WS group. Additionally, the correlation between reaction time and fixation-duration proportion for butterfly was significant for the MA group (*r* = −0.45). To establish reliable relationships, further studies that address these relationships should be conducted with more participants.

Here, we equalized all low-level visual information of the visual stimuli (e.g., luminance and size), and asked participants to detect a butterfly as quickly as possible. We did not mention that faces would also appear. Therefore, the butterfly should have been the most salient stimulus for the observers. Nevertheless, individuals with WS fixated on target-unrelated faces while searching for the target. This suggests that a face can be a more engaging stimulus in individuals with WS compared with typical controls. This atypical gaze has two explanations that are related to atypical explicit attentional control or visual attention processing captured by faces in individuals with WS. The first possibility is that prolonged fixation on a target-unrelated face is caused by atypical explicit attentional control of attentional shifting [23] or disengagement [24, 25]. Posner et al. [26] suggested that the cognitive act of shifting attention from one focus to another involves three distinct processes: disengagement of attention from the current focus, moving attention to a new target, and engagement of the new target. The dorsal visual attentional network controls such shifts and the allocation of endogenous visual attention [27]. Indeed, disengagement of attention may be a unique function of the parietal lobe [26]. Abnormalities in dorsal stream cortical structure may lead to the atypical face-related visual attention that we observed in individuals with WS [28]. The second explanation for the atypical gaze behaviors is that they are based on a problem with executive function that encompasses cognitive processes, such as attention set-shifting, working memory, and planning, that underlie goal-directed behavior [29]. This possibility is also supported by findings that people with WS have impaired response inhibition [9], perhaps arising from a failure to engage frontostriatal systems during inhibition tasks [30]. Poor executive function in individuals with WS, such as that required to sustain goal-directed behavior, might lead to atypical gaze behaviors toward target-unrelated upright faces.

As proposed by a useful model, face detection appears to be processed in the subcortical regions while face identification involves cortical regions, such as the fusiform gyrus [31]. The observed atypical preference for faces might result from irregular subcortical or cortical structures related to face processing. Consistent with this possibility, MRI studies have demonstrated abnormal subcortical and cortical volume in the thalamus and caudate of individuals with WS [32, 33]. Additionally, people with WS have been shown to have a larger amount of gray matter in the fusiform gyrus [32, 34], specifically the right fusiform gyrus [35], which includes fusiform face area (FFA). Moreover, individuals with WS exhibit enhanced neural activity in the FFA while viewing face stimuli compared with typical controls [36]. However, it remains unclear how these abnormal brain structures affect the faces preferences seen in individuals with WS. Further studies should address how atypical subcortical and cortical structures relate to face processing, perhaps by investigating the connection between the size of a subcortical or FFA region and atypical visual orientation in individuals with WS.

In addition to atypical fixation for upright faces, we found a significant negative correlation between RCPM scores (not chronological age) and the fixation duration for target-unrelated face stimuli. This was partly because the severity of the disorder is independent of age [37]. The current results raise further questions regarding how other cognitive abilities affect the amount of visual attention directed toward faces in individuals with WS. Further research should include larger samples to trace the developmental changes in visual attention toward faces and the underlying neural mechanisms in people with WS.

One limitation of our current study is that the ages of participants in the WS group were rather broad, including children and adults. As we have observed, some participants showed prolonged reaction time compared with others. We found that individual variability affects the relationship between eye movements and reaction time. In a further analysis, we divided the participants of CA and WS groups into young and adult groups based on their age. Results showed that performance accuracy and the first-fixation proportion were significantly affected by age. However, we did not observe any effect of age on reaction time or the fixation-duration proportion. These findings suggest that age alone does not fully explain the attentional profile found in our current experiment. Further studies are needed to trace how dynamic changes in allocating attention during the task change during development in young children with WS. Another issue is that although we revealed that visual attention is dominated by different factors (i.e., target-unrelated faces or the target) at different stages of searching, eye-movement measures are not sufficient for capturing detailed aspects of the attentional profile in individuals with WS. Further detailed analysis and more participants are needed to fully understand the dynamic aspect of the attentional allocation in the WS group.

## Conclusions

In conclusion, we have shown that the target-unrelated faces captured attention at the later stages of searching in individuals with WS. Furthermore, while manual responses are associated with gaze behavior in some aspects, compared with eye-movement data, they do not seem to be sensitive measures for characterizing the atypical attentional profile in people with WS in the current experimental paradigm. We propose that the current experimental setting and procedures have the potential for allowing reliable multimodal data, such as eye movements and manual responses, to be collected during visual attention tasks in individuals with and without developmental disorders. This procedure can provide insights into attentional processes in WS, particularly those related to faces, and is thus important in terms of understanding the everyday functioning atypicalities associated with this neurodevelopmental disorder.

## Abbreviations

ANOVA: Analysis of variance
AOI: Area of interest
CA: Chronological age-matched
MA: Mental age-matched
RCPM: Raven’s Colored Progressive Matrices test
WS: Williams syndrome
